# Colostrum and Mature Human Milk of Women from London, Moscow, and Verona: Determinants of Immune Composition 

**DOI:** 10.3390/nu8110695

**Published:** 2016-11-03

**Authors:** Daniel Munblit, Marina Treneva, Diego G. Peroni, Silvia Colicino, LiYan Chow, Shobana Dissanayeke, Priya Abrol, Shreya Sheth, Alexander Pampura, Attilio L. Boner, Donna T. Geddes, Robert J. Boyle, John O. Warner

**Affiliations:** 1 Department of Paediatrics, Imperial College London, London W2 1NY, UK; lychow8@gmail.com (L.C.); p.abrol@doctors.org.uk (P.A.); shreya.sheth10@imperial.ac.uk (S.S.); r.boyle@nhs.net (R.J.B.); j.o.warner@imperial.ac.uk (J.O.W.); 2 International Inflammation (in-FLAME) Network of the World Universities Network, Sydney 2006, NSW, Australia; trenevamarina@mail.ru (M.T.); diego.peroni@unipi.it (D.G.P.); apampura1@mail.ru (A.P.); donna.geddes@uwa.edu.au (D.T.G.); 3 Faculty of Pediatrics, I. M. Sechenov First Moscow State Medical University, Moscow 119991, Russia; 4 Allergy Department, Veltischev Clinical Pediatric Research Institute of Pirogov Russian National Research Medical University, Moscow 125412, Russia; 5 Department of Clinical and Experimental Medicine, Section of Paediatrics, University of Pisa, 56126 Pisa, Italy; 6 National Heart and Lung Institute, Imperial College London, London SW3 6NP, UK; s.colicino@imperial.ac.uk; 7 Royal Holloway University of London School of Biological Sciences, Biomedical Sciences, London TW20 0EX, UK; shobanadis@hotmail.com; 8 Department of Life and Reproduction Sciences, Section of Paediatrics, University of Verona, 37124 Verona, Italy; attilio.boner@univr.it; 9 School of Chemistry and Biochemistry, The University of Western Australia, Perth 6009, WA, Australia

**Keywords:** colostrum, human milk, immune modulators, immunologically active molecules, cytokines, growth factors, environmental influence

## Abstract

Cytokines and growth factors in colostrum and mature milk may play an important role in infant immune maturation, and may vary significantly between populations. We aimed to examine associations between environmental and maternal factors, and human milk (HM) cytokine and growth factor levels. We recruited 398 pregnant/lactating women in the United Kingdom, Russia, and Italy. Participants underwent skin prick testing, questionnaire interview, and colostrum and mature milk sampling. HM cytokine and growth factor levels were quantified by electro-chemiluminescence. We found significant geographical variation in growth factor levels, but no evidence of variation between sites in cytokine detectability. There was an inverse correlation between time of milk sampling and growth factor levels in colostrum for Hepatocyte Growth Factor (HGF) and TGFβ1 and TGFβ3, but not TGFβ2, and levels were significantly higher in colostrum than mature milk for all growth factors. The kinetics of decline were different for each growth factor. Cytokines were present at much lower levels than growth factors, and the decline over time was less consistent. HM growth factors and cytokine levels vary between populations for unknown reasons. Levels of HM mediators decline at different rates postpartum, and these findings suggest specific biological roles for HM growth factors and cytokines in early postnatal development.

## 1. Introduction

Human milk (HM) is usually the first source of nutrition for a newborn, and an important factor assisting infants in the first months of life, not only as a nutrition source but also as a manner of adapting infants to their extra-uterine environment. Breastfeeding is known to have positive short- and long-term effects on child health, although its influence on allergy development is still debatable [1]. There have been few attempts to investigate relationships between maternal and environmental factors and immune active profiles of HM. Data from a variety of studies suggest that factors such as country of maternal origin, diet, exercise, and exposure to smoke or farming environment in early life may influence colostrum and HM constituents [2,3,4,5,6,7,8,9,10,11,12,13,14,15,16,17,18,19,20,21,22].

It has been shown that Hepatocyte Growth Factor (HGF) is known to regulate vascular endothelial growth factor (VEGF) production from endothelial cells [23] and complements VEGF biological activity in the infant gut [24]. HGF levels in maternal serum change throughout pregnancy, peaking at 30 to 40 weeks of gestation and then decreasing upon delivery [25]. As levels of HGF are 20 to 30 times higher in colostrum than in maternal serum [26] it is reasonable to propose that HGF is actively excreted into HM in order to support infant’s gut immunity maturation and growth. 

Inverse relationships between the risk of atopic diseases, associated with T-helper lymphocyte type 2 (TH2) immune response, and indicators of early-life exposure to infections, such as high birth order or sibship size, early attendance at day care, and early exposure to pets or other animals are well described [27]. The “hygiene hypothesis” remains one of the most popular current hypotheses on early-life exposures and allergy risk [28]. It has been suggested that farming environment [6], higher bacterial exposure [7], or maternal country of origin [5] may have significant impacts on HM immune composition, and such effects on HM composition may be an important pathway through which early variations in microbial exposures influence risk of allergy development.

Our study aimed to prospectively investigate the relationship between maternal and environmental factors and levels of HGF, Transforming Growth Factor beta (TGFβ)1,2,3 and detectability of TH1 and TH2 cytokines using colostrum and HM samples collected from birth cohorts in three regions; the UK, eastern Europe, and continental Europe. 

## 2. Materials and Methods 

### 2.1. Study Setting, Eligibility Criteria, and Ethics

The investigations and sample collection have been conducted following ethical approval by Ethics committee in three countries participating in the study: West London Rec 3 (UK) (Ref. number 10/H0706/32) and all paperwork has been completed according to the hospital R&D Joint Research Office (UK) (JROSM0072) policy; Ethical Committee of the Azienda Ospedaliera di Verona (Italy) (approval No. 1288), and Moscow Institute of Paediatrics and Child Health of Ministry of Health of Russian Federation (Russia) (approval No. 1-MS/11). All women provided written informed consent.

Women were enrolled at antenatal and postnatal units of three participating centres—St. Mary’s Hospital, London, UK; Maternity Hospital No. 1, Moscow, Russia; G.B. Rossi Hospital, Verona, Italy. Inclusion criteria for the study were: healthy term infants and their mothers intending to breastfeed and willing to comply with the study procedures. 

Exclusion criteria were: maternal immunosuppressive treatment during lactation, or severe illness; infants with a major birth defect, admitted to neonatal intensive care, other severe illness, born prematurely (<37 weeks gestation), or with low birth weight (<2nd centile). 

### 2.2. Medical Records and Interview

Following enrolment, participants underwent allergy skin prick testing (SPT) and answered a 10 min interview-based questionnaire regarding their medical history. Exposure variables recorded were selected based on a detailed review of known determinants of HM composition [29]. Information collected from the recruited women included parity; age; mode of delivery; details of residence environment, such as mould presence at home, regular contact with animals and/or pets at home; exposure to tobacco smoke (smoker or living in household with smoker or self-reported passive smoker); any reports of infections during pregnancy. We also obtained information on maternal dietary preferences—fish, fresh fruit, and probiotic intake. Participant medical records were reviewed by study personnel to extract relevant health information which was not available from questionnaires, prior to breast milk analysis. SPT was undertaken using the following solutions: Histamine 1% Positive Control, Glycerol Negative Control, House Dust Mite (*Dermatophagoides pteronyssinus*), Cat (*Felix domesticus*), Grass Pollen, Birch pollen, Peanut, Hazelnut, Egg (all from Stallergenes, SA 92160 Anthony, France), and Cow’s milk (ALK-Abello, Hǿrsholm, Denmark). SPT was performed by standard technique using 1 mm lancets (ALK-Abello, Hǿrsholm, Denmark), and were read at 15 min. Allergic sensitization was defined as a wheal ≥3 mm to at least one allergen, in the context of a wheal ≥3 mm to histamine and no wheal to the negative control.

### 2.3. Human Milk Sampling 

Participants were given sterile tubes to collect their own colostrum (once in the first 6 days of life) and mature HM (once at 4–6 weeks postpartum). Local site investigators and participating mothers were asked to collect colostrum or milk at the first morning breastfeed, by manual expression (colostrum) or collecting the drip (mature milk) from the contra-lateral breast during feeding [15]. Colostrum samples were frozen at −50 °C to −80 °C within 12 h of collection. HM samples were collected at home, stored in the fridge for not longer than 4 h, and transported to participating units by study staff, and frozen at 50 °C to −80 °C within 12 h of collection. It has been previously demonstrated that storage for 6 months at either −20 °C or −80 °C did not influence the concentration of immune active factors in human milk [30]. After thawing, samples were centrifuged at 1500× *g* for 15 min at 4 °C. The lipid layer was removed with a pipette and aqueous fraction was analysed for immune modulators [31]. All milk samples were transported to London at −70 °C where the samples were stored at −80 °C until analysis.

### 2.4. Electro-Chemiluminescence

We used electro-chemiluminescence to measure immune mediators in colostrum and breast milk samples for Th1 and Th2 cytokines, HGF, and TGFβ1-3 (MesoScale Discovery, Rockville, MD, USA). Laboratory experiments were run according to manufacturer’s protocol, using an eight-point standard curve. No dilution was used for Th1 and Th2 cytokines and HGF, and 1:2 dilution for TGFβ assays, following pilot experiments which showed that TGFβ2 levels in undiluted milk samples were often greater than the upper limit of detection. Assays were run in duplicate, and mediator levels were excluded where the CV was >25%—Median (IQR) levels and assay detection limits for each immune mediator studied are shown in Table A1. 

### 2.5. Protein Analysis 

We used turbidimetry to assess total protein concentration in colostrum samples (Abbott Architect Analyser C8000, Abbott, Abbott Park, IL, USA). For turbidimetry colostrum, proteins were denatured by benzethonium chloride, then measured at 404 nm. Pilot experiments determined that a 1:30 onboard dilution was needed to bring the colostrum protein concentration to within the linear measurement range of the Architect Analyser (3–60 g/L). Prior to protein analysis samples were thawed and centrifuged at 3000× *g* for 15 min at 4 °C. Next, 300 μL of supernatant were carefully transferred to the tubes and loaded into the Architect device for the analysis. 

### 2.6. Statistical Analysis

Maternal factors and levels/detection of cytokines and growth factors were summarized using standard descriptive statistics. As outcome variables were not normally distributed, non-parametric tests such as the Mann–Whitney *U*-test, were used to compare independent observations of different populations for unadjusted analyses. All growth factors have been presented as a continuous variable (pg/mL), whilst data on cytokines were transformed into a binary variable (detectable versus undetectable). Since concentration of growth factors and cytokines were assayed twice (in both colostrum and breast milk) for many participants, we used a mixed-effect regression model to account for the absence of independence between those two measures and to evaluate a broad range of variables. Continuous data with non-parametric distribution were log-transformed for inclusion in regression models.

Model selection was based on major approaches such as Akaike and Bayesian information criteria. Difference between groups effects (maternal and environmental factors) as well as within subject effects were evaluated using multilevel mixed-effect regression model. Factors and covariates included in the models were: parity, maternal sensitisation, maternal age, site of collection, mode of delivery—vaginal birth/caesarean section, mould presence at home, pets at home or regular contact; exposure to tobacco smoke (i.e., maternal active smoking or living in household with smoker or self-reported passive smoker) at recruitment; at least one self-reported maternal infection during pregnancy; maternal diet—fish intake at least once per week versus less often; daily fresh fruit versus less often; daily probiotic versus none/less often. 

As models were used for the statistical analysis, the sample size reduced slightly, due to incomplete data for one or more variables included in the multivariate model in 56 individuals. This explains discrepancy in colostrum numbers. Reduced numbers were included in analysis of human milk samples, due to missing samples. Milk samples were missing due to cessation of breastfeeding before this time, due to mother’s not supplying a sample within the four to six week time window for sampling, and due to loss to follow up/not contactable women.

Outcomes assessed were levels of HGF, TGFβ1, TGFβ2, TGFβ3, and cytokine detectability. Relationship between corrected growth factor levels and time (measured as HGF/Protein, TGFβ1/Protein, TGFβ2/Protein, and TGFβ3/Protein in pg/g) was assessed using the Spearman rank correlation coefficient. Results were considered significant when p-values were reported at a level less than 0.05. Bonferroni correction was used in mixed models analyses to control for false discovery. 

## 3. Results

### 3.1. Study Population

Total of 481 mothers were recruited into the study from June 2011 to March 2012 from the birth centres and antenatal and postnatal units of secondary and tertiary hospitals from three countries, located in Northern Europe, Eastern Europe, and the Mediterranean area. Of 481 women, 398 (UK *n* = 101, Russia *n* = 221, Italy *n* = 76) provided samples and were included in this study. The 83 mothers unwilling or unable to provide colostrum samples postnatally were not evaluated further.

Demographic data of the participants is presented in Table 1. Significant differences between groups were seen for most variables recorded. Maternal age (highest in Italy), maternal allergic sensitization (highest in UK), rate of Caesarean section (highest in UK), tobacco smoke exposure (highest in Russia), antenatal infections (highest in Italy), and time of colostrum collection (earlier in Russia than other sites) all differed significantly. There was also weak evidence for differences in parity and birth weight across centres). Infant sex did not significantly differ across sites.

### 3.2. Association between Collection Time and Colostrum/Breast Milk Composition

**Figure 1:**
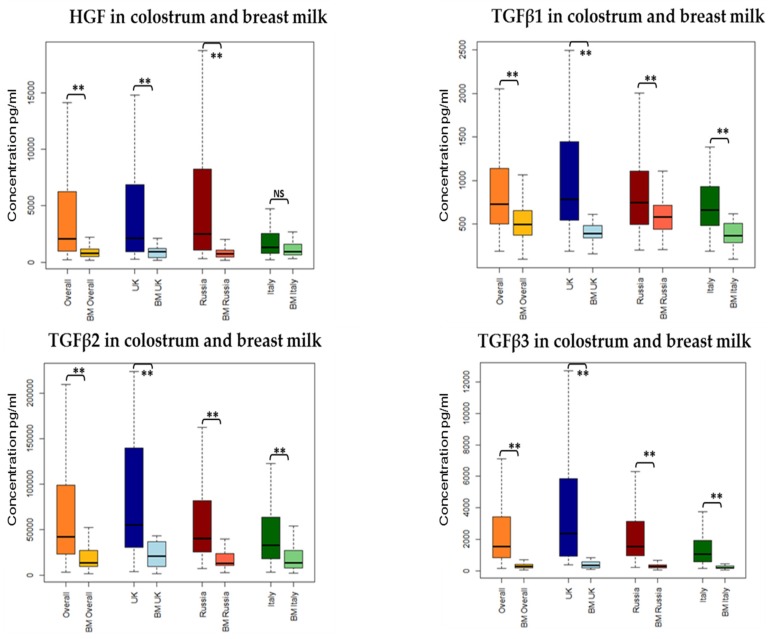
Unadjusted growth factors concentration (pg/mL) in colostrum and breast milk across all sites (overall) and at each site of collection (London, Moscow, and Verona). ** *p* value < 0.01.

Among the environmental and maternal factors analysed we found time of milk sample collection postpartum to have the most significant influence on growth factor levels, and a mixed effect on cytokine detectability. A significant decline over time in colostrum was seen for HGF, TGFβ1 and TGFβ3, IL2, IL5, IL10, and IFNγ. No significant influence but similar trends were seen for most of the other mediators (Table 2 and Figure 1). Differences between colostrum and mature milk showed similar findings for growth factors, which were all statistically significant. For cytokine detectability in breast milk versus colostrum, findings were mixed and not entirely consistent with the changes seen over time in colostrum. IL10 was more commonly detected in colostrum than in HM, but IL4 and IFNγ were more commonly detected in HM, despite no change or a decline in detectability over time in colostrum samples. Other cytokines showed no significant difference between colostrum and HM (Table 2).

### 3.3. Association between Collection Site and Colostrum/Breast Milk Composition

#### 3.3.1. Growth Factors

**Figure 2:**
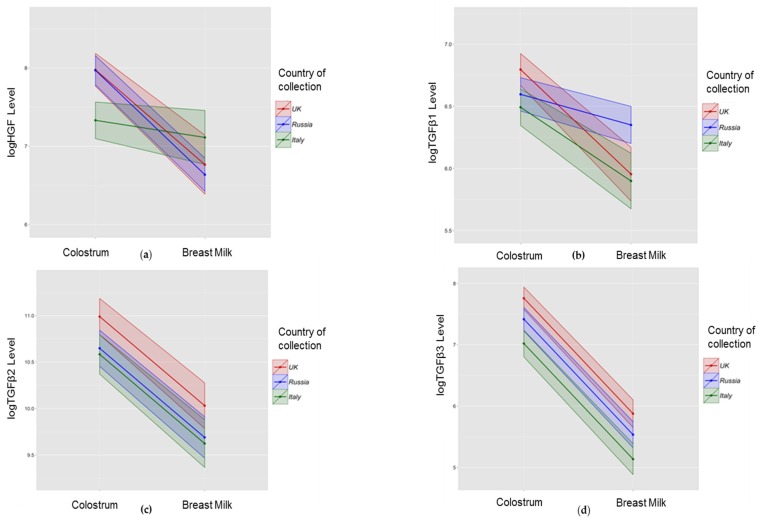
Association between site of collection and growth factor concentrations in colostrum and breast milk. Data shown are mean (bold line) and 95% CI (shaded area) for log transformed concentrations of HGF (**a**); TGFβ1 (**b**); TGFβ2 (**c**); and TGFβ3 (**d**) in the UK (**red**), Russia (**blue**), and Italy (**green**). A multilevel mixed-effect regression model was used for all analyses which were adjusted to the following factors: Parity, Maternal Atopy, Maternal age, Site (Country) of collection, Mode of delivery, Mould presence at home, Pets at home or regular contact; Exposure to tobacco smoke, at recruitment; At least one self-reported maternal infection during pregnancy; Maternal diet.

We found a significant influence of country of residence on some growth factor concentrations (Table 3 and Figure 2). TGFβ2 and TGFβ3 showed consistent findings in colostrum and HM samples—levels of both factors were higher in UK and lower in Italy, with intermediate levels in Russian women. HGF was lowest in the colostrum, but not HM, of mothers in Italy (mean log HGF 7.38, SE 0.12 in Italy; 7.98 (0.11) UK; 7.99 (0.10) Russia). TGFβ1 did not show consistent differences between sites in colostrum and HM analyses, with higher levels in UK colostrum samples compared with Italy (mean log TGFβ1 6.80 (0.07) in the UK; 6.53 (0.08) Italy); and high levels in Russian HM samples (6.36 (0.08)) compared with Italy (5.86 (0.11)) or the UK (5.96 (0.11)). We evaluated a number of other maternal factors for association with growth factor concentrations, and found weak evidence of an association between fish consumption less than once a week and higher levels of TGFβ1 (mean log TGFβ1 6.72 (0.06) for less than once a week; 6.57 (0.05) for more than once a week); and primiparity with higher levels of HGF in colostrum (7.87 (0.08) for primiparous; 7.69 (0.08) for multiparous) and HM (6.92 (0.11) for primiparous; 6.75 (0.11) for multiparous).

#### 3.3.2. Cytokines

Women reporting infections during pregnancy had detectable levels of IL5 less often in comparison to mothers reporting no antenatal infections (Table 4). Smoking, type of delivery, and antenatal infections were not related to detection of cytokines in HM or colostrum.

#### 3.3.3. Protein and Sodium in Colostrum

**Figure 3:**
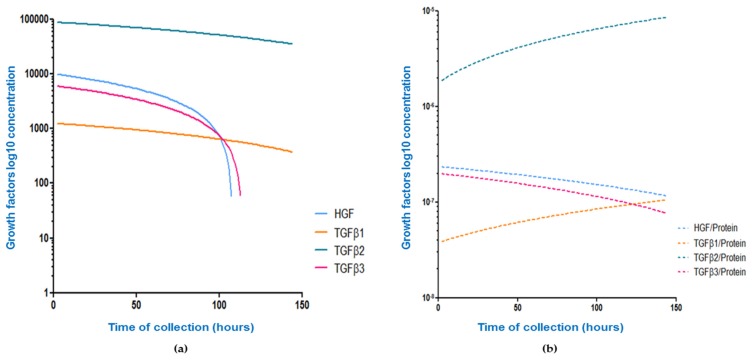
Effect of correcting growth factor concentrations for total protein level on the relationship between concentration and time. Data show nonlinear curves for unadjusted concentrations (pg/mL) of HGF (**blue**), TGFβ1 (**orange**), TGFβ2 (**green**), and TGFβ3 (**pink**) in colostrum in relation to time of sampling. Correlations were explored using raw data (**a**); and the ratio of growth factor concentration to protein concentration in the same sample (**b**). Trends in absolute and relative growth factors concentration in colostrum change over time. All correlation coefficients were statistically significant apart from the TGFβ3/Protein ratio in Figure 3b.

Protein in human colostrum declines over time (*r* = −0.42; *p* < 0.001) with all growth factor levels demonstrating a significant and marked decline over time, although the slope of decline varied between growth factors. When corrected for protein, the correlation between growth factor levels and time of collection was inconsistent between factors, with a significant decline seen for HGF/Protein, an increase over time seen for TGFβ1/Protein and TGFβ2/Protein, and no change over time seen for TGFβ3/Protein. The relationship between time of colostrum collection and growth factor or growth factor/Protein level for the four growth factors measured is shown in Table A2 and Figure 3.

## 4. Discussion

In this large prospective cohort study, we have confirmed earlier findings of significant differences in milk composition between sites, but were not able to explain these differences through maternal or environmental factors. We identified important differences between mediators in kinetics of decline postpartum and these data suggest specific mechanisms controlling HM immune composition, and support important biological roles for HM immune factors in the developing infant.

Data from a variety of studies suggest that colostrum and HM constituents may be influenced by the country of origin and a number of environmental conditions which may differ significantly from one location to another [3,4,5,6,7,32], but the cause of this difference is still unclear. Our data suggest that HGF levels were lower in the colostrum/milk of Italian mothers and TGFβ2 and TGFβ3 were higher in colostrum/milk of UK mothers with the same significant difference for colostral TGFβ1 (Figure 2). Amoudruz showed that Mali women have higher levels of TGF-β1 in comparison with women born in Sweden [5]. Peroni reported higher HM TGF-β1 in a farming compared with urban environment [6]. Tomicic found Estonian mothers have lower HM TGFβ2 than Swedish mothers [7] which the authors suggested may be due to differences in microbial exposure. Orivuori et al. assessed HM samples collected in four countries of continental Europe and Finland and showed TGFβ1 levels to be highest in Finland and sIgA lowest in Germany [32]. We explored a number of maternal and demographic variables including markers of microbial load such as maternal report of probiotic, pet, and mould exposure, but we were not able to confirm these previous findings. 

At present, we do not possess strong evidence of maternal allergic status influences on qualitative and quantitative immunological constituents in HM. We did not find any relationship between the levels of growth factors and/or detectability of cytokines and maternal allergy; this is in agreement with data from other studies which found no obvious trend in HM composition of allergic mothers compared to non-allergic [32,33,34]. 

Our data show some evidence for higher levels of HGF and TGFβ3 in the colostrum of primiparous women. Data from some studies suggest that parity does not influence HM composition. This borderline trend can be seen in some (but not all [3,35]) other studies [4,36,37], suggesting that higher levels of certain immune active markers can be found in the HM of primiparous mothers, which may be an additional mechanism to explain decreased allergy risk with an increase of birth order.

We found some evidence that fish intake during pregnancy can influence HM composition [3]—we found higher colostrum TGFβ1 in women eating fish less than once a week. This adds to a confusing picture, with Urwin reporting TGF-β1 levels to be highest in the colostrum of women residing in the river and lake region of China [3], well known for high fish consumption and Hawkes not finding any relationship between fish oil intervention and TGFβ1 levels [38]. Thus, the reasons behind differences in the HM immunological profile between countries remain unclear. Further work should consider genetic and gene/environment influences, as well as detailed dietary assessments, in addition to the factors evaluated in this study. Data from So-Yeon Lee and co-authors show that breastfeeding was found to be associated with a reduced risk of allergic sensitisation in children with CT/CC geno-type, suggesting gene-environment interaction between the CD14C-159T polymorphism and breastfeeding in relation to aeroallergen sensitisation [39].

It is well established that colostrum is particularly rich in immunologically active molecules and that levels of immunological factors are lower in mature HM [6,12,40,41]. Although our study did not involve HM collection in the same individuals over multiple timepoints, the sample size was large enough to identify a strong relationship between time of sampling postpartum and growth factor levels. We observed a strong relationship between growth factor levels and time postpartum both within colostrum, and between colostrum and mature milk samples, which was independent of geographical location. Although the difference between colostrum and mature milk composition is well established in the literature [6,12,40,41], our finding of a rapid decline in growth factor levels (HGF, TGFβ1, TGFβ3) and evidence for reduced detectability of cytokines (IL2, IL5, IL10, IFNγ) in colostrum with time postpartum has not been consistently demonstrated by others. Soto-Ramirez et al. found none of the immune markers to correlate with the time of milk collection in a study conducted in the USA [42]; this may be partially explained by a wide range in collection times (week one to eight) and absence of colostral samples. In a study done in China with a narrower range of collection times, levels of TGF-β1 and TGF-β2 decreased significantly over time [3]. Studies which do not adequately account for variations in sampling time may explain some of the inconsistency of HM composition and its determinants in the literature [29]. 

Dilution could be a potential explanation of the growth factor decline over time. During the first week of life the infant’s volume requirements are low. Later levels of the immune active molecules decrease as the volume and nutritional requirements of the infant increase. Immune active constituents of colostrum and breast milk represent a minor component but one of the most biologically active parts of HM total protein. In this study, we attempted to adjust for dilutional effects by correcting growth factor colostrum levels for total protein level in the same sample. Correction for colostrum total protein failed to consistently remove the relationship between factor level and time postpartum, and highlighted significant variation between factors in the kinetics of decline. This implies active transport of these growth factors using varied mechanisms specific to each factor. If we assume biological relevance, then these results suggest that infants need relatively higher amounts of TGFβ1 and TGFβ2 for longer than that of TGFβ3 or HGF. TGFβ3 is significantly different from TGFβ1 and TGFβ2 in its detailed tertiary structure of the active domain despite homology in amino acid sequence [43]. There is some evidence [43] that TGFβ3 may also have distinct functions to other TGFβ isoforms. TGFβ3 is up-regulated by milk stasis, and induces apoptosis in mammary gland epithelium during involution, in contrast to TGFβ1 and TGFβ2 [44]. The biological relevance of HM TGFβ is illustrated by the observed direct correlation between the levels of TGFβ in human milk and infant serum IgA [45]. As the infant’s immune response matures there is likely to be less need for an extrinsic supply of immune stimulants.

The main limitations of our study are first that we did not assess maternal diet using a food frequency questionnaire, did not collect detailed information on the strain of probiotics and/or fish oil supplements used, and did not evaluate maternal genotype as a potentially important determinant or modulator of HM composition. This means that while we were able to explore our primary focus of maternal and demographic factors influence on HM composition, we were not able to reliably identify reasons for the difference in HM composition identified between sites. Second, we sampled single colostrum and HM specimens from each subject. Our findings regarding the kinetics of growth factor decline in colostrum and HM need to be explored further in prospective studies with longitudinal sampling at multiple timepoints within the same participants. Third, we only made preliminary attempts to adjust for the effect of time or milk maturity on compositional analysis. Since time postpartum may be a surrogate for milk/breast maturity, we aimed to identify a ‘correction factor’ that might reflect milk maturity. We did not find evidence that any growth factor concentration was related to HM protein concentration, but further work is needed to identify markers of HM maturity in order to control for this in compositional analysis of colostrum. Maternal body mass index (BMI) data has not been assessed due to a difficulty in one of the participating countries. Finally, due to logistical, funding, and sample size constraints our analysis covers only a small number of growth factors and cytokines, so may not be generalisable to all immune factors in HM.

## 5. Conclusions 

In this large international cohort study of HM composition, we have found an important influence of time after birth on colostrum composition, which must be adjusted for in any further research. Despite adjusting for this and other factors, we identified unexplained and significant variation in HM immune composition between geographically distant populations. Overall, our data suggest that levels of human milk mediators decline at different rates post-partum, and this may suggest that these factors are likely to have specific biological roles in early postnatal development. An important aim of future research should be to optimize the analysis of HM composition, accounting for maturity of milk, and aim to better understand the biological roles and consequences for the developing infant of variations in HM immune composition.

## Figures and Tables

**Table 1:** Characteristics of study participants.

	UK	Russia	Italy	*p-*Value (Three Countries)
Maternal allergic sensitisation *	35/94 (37)	22/156 (14)	9/40 (23)	<0.01 ^a^
Maternal Age (years)	32.8 (4.78)	29.8 (4.45)	37.4 (5.38)	<0.01 ^b^
Vaginal Delivery	70/101 (69)	188/219 (86)	62/76 (82)	<0.01 ^a^
Male sex	54/101 (53)	118/216 (55)	41/76 (54)	0.98 ^a^
Birth Weight (grams)	3527 (535.37)	3526 (438.97)	3328 (476.95)	0.05 ^b^
Primiparous women	55/100 (55)	93/216 (43)	29/75 (39)	0.06 ^a^
Household tobacco smoke exposure	30/99 (30)	135/218 (62)	25/76 (33)	<0.01 ^a^
Antenatal Infections ^†^	16/100 (16)	61/211 (29)	29/76 (38)	<0.01 ^a^
Time of colostrum collection (hours)	58.61 (33.2)	50.03 (14.34)	57.84 (26.52)	<0.01 ^b^

^a^ Pearson χ^2^ test has been used; ^b^ ANOVA test has been used. Data shown are (*n*/(%)) for binary variables, and (mean (S.D.)) for continuous variables; * Defined as skin prick test wheal ≥3 mm to at least one of a panel of common allergens; ^†^ Antenatal infection is defined as at least one self-reported maternal infection during pregnancy.

**Table 2:** Relationship between time of sample collection and milk composition.

Immune Modulator	Colostrum Composition			Paired Differences between HM and Colostrum of Colostrum and Breast Milk Composition		
	Change over Time	β	*p*-Value	Difference between Colostrum and HM over Time	β	*p*-Value
**HGF**	Lower	−0.01	<0.001 **	Higher in colostrum	−1.35	<0.001 **
**TGFβ1**	Lower	−0.003	0.01 *	Higher in colostrum	−0.93	<0.001 **
**TGFβ2**	No change	−0.003	0.12	Higher in colostrum	−1.12	<0.001 **
**TGFβ3**	Lower	−0.01	<0.001 **	Higher in colostrum	−2.03	<0.001 **
**IL2**	Lower	−0.02	0.02 *	No difference	0.32	0.30
**IL4**	No change	−0.01	0.22	Higher in HM	0.72	0.04 *
**IL5**	Lower	−0.03	<0.001 **	No difference	−0.54	0.09
**IL10**	Lower	−0.02	<0.001 **	Higher in colostrum	−1.66	<0.001 **
**IFNγ**	Lower	−0.01	0.04 *	Higher in HM	1.20	<0.001 **
**IL12**	No change	−0.01	0.12	No difference	−0.11	0.73
**IL13**	No change	−0.01	0.09	No difference	0.09	0.72

Data shown are concentration (pg/mL) for growth factors, and detectable versus not detectable for cytokines. A multilevel mixed-effect regression model was used for all analyses, which were adjusted to the following factors: Parity, Maternal Atopy, Maternal age, Site of collection, Mode of delivery, Mould presence at home, Pets at home or regular contact; Exposure to tobacco smoke; At least one self-reported maternal infection during pregnancy; Maternal diet. HM, human milk; HGF, Hepatocyte Growth Factor. * *p* value < 0.05; ** *p* value < 0.01.

**Table 3:** Concentration (pg/mL) of growth factors in colostrum and breast milk and exposures associated with the levels.

	Median (IQR) pg/mL	Important Growth Factor Level Difference between the Groups	
Colostrum			
HGF	2055.31 (964–6239)	UK and Russia higher than in Italy	*p* < 0.001
		Primipara higher than Multipara	*p* = 0.05
TGFβ1	731.534 (505–1142)	UK higher than Italy	*p* = 0.01
		Fish consumption Less than once a week higher than At least once a week	*p* = 0.04
TGFβ2	42,209.88 (23,847–98,597)	UK higher than Russia and Italy	*p* < 0.05
TGFβ3	1535.081 (847–3395)	UK higher than Russia higher than Italy	*p* < 0.05
Breast Milk			
HGF	784.041 (508–1189)	Primiparous higher than Multigravida	*p* = 0.05
TGFβ1	493.514 (375–653)	Russia higher than UK and Italy	*p* < 0.05
TGFβ2	14,040.62 (10,080–27,262)	UK higher than Russia and Italy	*p* < 0.05
TGFβ3	279.41 (183–395)	UK higher than Russia higher than Italy	*p* < 0.05

A multilevel mixed-effect regression model was used for all analyses, which was adjusted using Bonferroni correction, to the following factors: Parity, Maternal Atopy, Maternal age, Site (Country) of collection, Mode of delivery—labour versus no labour, Mould presence at home, Pets at home or regular contact; Exposure to tobacco smoke (i.e., smoker or living in household with smoker or self-reported passive smoker) at recruitment; At least one self-reported maternal infection during pregnancy; Maternal diet—fish intake at least once per week versus less often; daily fresh fruit versus less often; daily probiotic versus none/less often.

**Table 4:** Detectability of Th1 and Th2 Cytokines in Colostrum and Human Milk.

	Colostrum Detectable	Human Milk Detectable	Factors Associated with Cytokines Detectability
IL2	49/342 (14%)	38/190 (20%)	NA
IL4	35/342 (10%)	30/190 (16%)	NA
IL5	77/342 (23%)	27/190 (14%)	Antenatal infections OR 0.49 (95% CI 0.25–0.98)
IL10	225/342 (66%)	69/190 (36%)	NA
IFNγ	66/342 (19%)	92/190 (48%)	NA
IL12	63/342 (18%)	31/190 (16%)	NA
IL13	86/342 (25%)	58/190 (31%)	NA

NA—no association. A multilevel mixed-effect regression model was used for all analyses which were adjusted to the following factors: Parity, Maternal Atopy, Maternal age, Site (Country) of collection, Mode of delivery, Mould presence at home, Pets at home or regular contact; Exposure to tobacco smoke, at recruitment; At least one self-reported maternal infection during pregnancy; Maternal diet.

## Appendix A

**Table A1:** Lower limit of detection values (pg/mL) for cytokines and growth factors analysis and overall locations median concentrations for all immune active molecules (values below LLOD for all cytokines have been transformed into ½ of LLOD).

Immune Active Molecule	Median Lower Limit of Detection	Median (IQR) Colostrum	Median (IQR) HM
IFN-γ	3.49	1.23 (0.51–3.49)	3.49 (1.54–7.8)
IL2	2.06	0.6 (0.29–1.47)	0.98 (0.23–2.06)
IL4	1.83	1.83 (0.24–1.83)	1.83 (0.50–1.83)
IL5	2.89	1.44 (0.57–2.85)	1.57 (0.33–2.89)
IL10	1.50	2.67 (1.01–9.04)	1.5 (0.74–2.66)
IL12	3.50	1.85 (0.34–3.5)	2.13 (0.32–3.5)
IL13	4.60	4.6 (1.85–4.69)	4.6 (3.52–6.76)
HGF	73.00	2055.31 (964–6239)	784.041 (508–1189)
TGFβ1	8.73	731.534 (505–1142)	493.514 (375–653)
TGFβ2	265.00	42,209.88 (23,847–98,597)	14,040.62 (10,080–27,262)
TGFβ3	8.37	1535.081 (847–3395)	279.41 (183–395)

Standard curve ranges: IFN-γ, IL2, IL4, IL5, IL10, IL12, IL13 0—2500 pg/mL; HGF, TGFβ1, TGFβ2 0—100.000; TGFβ3 0—50.000. All values presented in pg/mL.

**Table A2:** Absolute and relative decline of growth factors over time.

Growth Factor Concentration (pg/mL)/Time of Collection	Raw Concentration (pg/mL)	Growth Factor/Protein Ratio
**HGF**	*r* = −0.39, *p* < 0.001 ↓	*r* = −0.19, *p* = 0.003 ↓
**TGFβ1 **	*r* = −0.21, *p* < 0.001 ↓	*r* = 0.25, *p* < 0.001 ↑
**TGFβ2 **	*r* = −0.16, *p* = 0.01 ↓	*r* = 0.20, *p* = 0.004 ↑
**TGFβ3 **	*r* = −0.35, *p* < 0.001 ↓	*r* = −0.06, *p* = 0.34 ↓

↑—Positive correlation with time of collection; ↓—Negative correlation with time of collection.
